# Animal models in the study of diabetic erectile dysfunction: mechanisms and applications

**DOI:** 10.3389/fendo.2025.1512360

**Published:** 2025-03-24

**Authors:** Xin Zhang, Yihao Chen, Jiahua Qian, Yuhe Si, Chenxi Wang, Jingwei Wang, Qiang He, Jianxiong Ma

**Affiliations:** ^1^ The Second Clinical Medical College, Zhejiang Chinese Medical University, Hangzhou, Zhejiang, China; ^2^ The First Clinical Medical College, Zhejiang Chinese Medical University, Hangzhou, Zhejiang, China; ^3^ Academy of Chinese Medical Sciences, Zhejiang Chinese Medical University, Hangzhou, Zhejiang, China; ^4^ Department of Nephrology, the First Affiliated Hospital of Zhejiang Chinese Medical University (Zhejiang Provincial Hospital of Chinese Medicine), Hangzhou, Zhejiang, China; ^5^ Zhejiang Key Laboratory of Research and Translation for Kidney Deficiency-Stasis-Turbidity Disease, Zhejiang Chinese Medical University, Hangzhou, Zhejiang, China

**Keywords:** diabetic erectile dysfunction, animal models, rodent animals, pathogenesis, treatment strategies

## Abstract

**Background:**

Diabetic erectile dysfunction (DMED) is a common complication of diabetes. While research on DMED relies primarily on animal models, replicating the intricate etiology and multi-system interactions of human DMED in a single model remains a challenge.

**Aim:**

This article provides a comprehensive overview of animal models used in DMED research and emphasizes the crucial role they play in understanding the pathogenesis and treatment of DMED.

**Methods:**

A comprehensive medical literature was searched in PubMed and Medline, focusing on original studies and systematic reviews of original studies involving animal models of diabetic erectile dysfunction. Clinical studies, editorials, letters, reviews, and non-English articles were excluded.

**Results:**

This article compiles various animal models currently used in the study of diabetes and diabetic erectile dysfunction (DMED), with a particular emphasis on the application of rodent models such as rats and mice. These animals demonstrate significant advantages in terms of economy, practicality, and reproducibility in DMED research and share similarities with humans in tissue morphology and functional characteristics.

**Conclusion:**

This manuscript offers researchers multiple insights into selecting animal models for DMED, particularly considering their practicality, cost-effectiveness, and reproducibility. The integrated information serves as a valuable reference for researchers in choosing suitable models.

## Introduction

In recent years, the global prevalence of diabetes mellitus (DM) has witnessed exponential growth. According to statistics, in 2019, approximately 463 million people worldwide were affected by DM (1). By 2040, this number will increase to 640 million among people aged 20-79 (2). Erectile dysfunction (ED) is a frequent complication of type 1 diabetes (T1DM) and type 2 diabetes (T2DM). Epidemiological surveys indicate that ED affects diabetic patients at an incidence ranging from 19.5% to 75.2% (3). Numerous studies have demonstrated that the incidence of ED is approximately 3.5 times higher in DM patients compared to non-diabetic individuals (4) ED tends to manifest in DM patients approximately 10 years earlier than in the general population (5). Its pathogenesis involves intricate physiological changes encompassing factors like nerve, blood vessels, muscle, and gonadal axis dysfunction or decline. Currently, most research on DMED is carried out using animal models. Given the complexity of DMED, particularly the psychosomatic and multi-systemic interactions, animal models serve as indispensable tools in dissecting the underlying physiological mechanisms that are often challenging to study in human patients. This review aims to elucidate how animal models contribute not only to understanding the vascular and neural pathways involved in ED but also to exploring potential therapeutic interventions. The practice of employing modern, standardized animal models for diabetes dates back to the 17th century when Bruner first pioneered it. Later, in the 19th century, Claude Bernard made significant strides in the field through his experiments *in vivo*, which utilized animal models to explore the endocrine and exocrine functions of the pancreas. While there are various animal models available for diabetes research, none can completely simulate the complex pathogenesis and multi-system interactions found within the human body.

Thus, the development of a rational, practical, and scientifically grounded animal model for DMED is essential to gain a profound comprehension of its pathogenesis and resulting secondary effects. In previous work, we utilized streptozotocin (STZ) injection to establish the well-known rat model of DMED, aiming to investigate the effectiveness and underlying mechanisms of drug treatments, resulting in valuable advancements (6–8). This article is a component of our ongoing project on DMED animal models, offering a comprehensive compilation of diverse DM animal models extensively employed in contemporary research, serving as a valuable guide for the selection process.

## Methods

The medical literature was searched in PubMed and Medline to identify peer-reviewed original studies and systematic reviews on animal models of diabetic erectile dysfunction. The following keywords were used in various combinations: “diabetes”, “erectile dysfunction”, “erectile function”, “sexual dysfunction”, “diabetic erectile dysfunction”, “animals”, “animal models”. ***Inclusion Criteria:*** Animal studies related to diabetic erectile dysfunction. ***Exclusion Criteria:*** Non-original research literature such as editorials, letters, conference abstracts, and commentaries; Studies solely involving human clinical research; Articles published in languages other than English.

**Figure 1:**
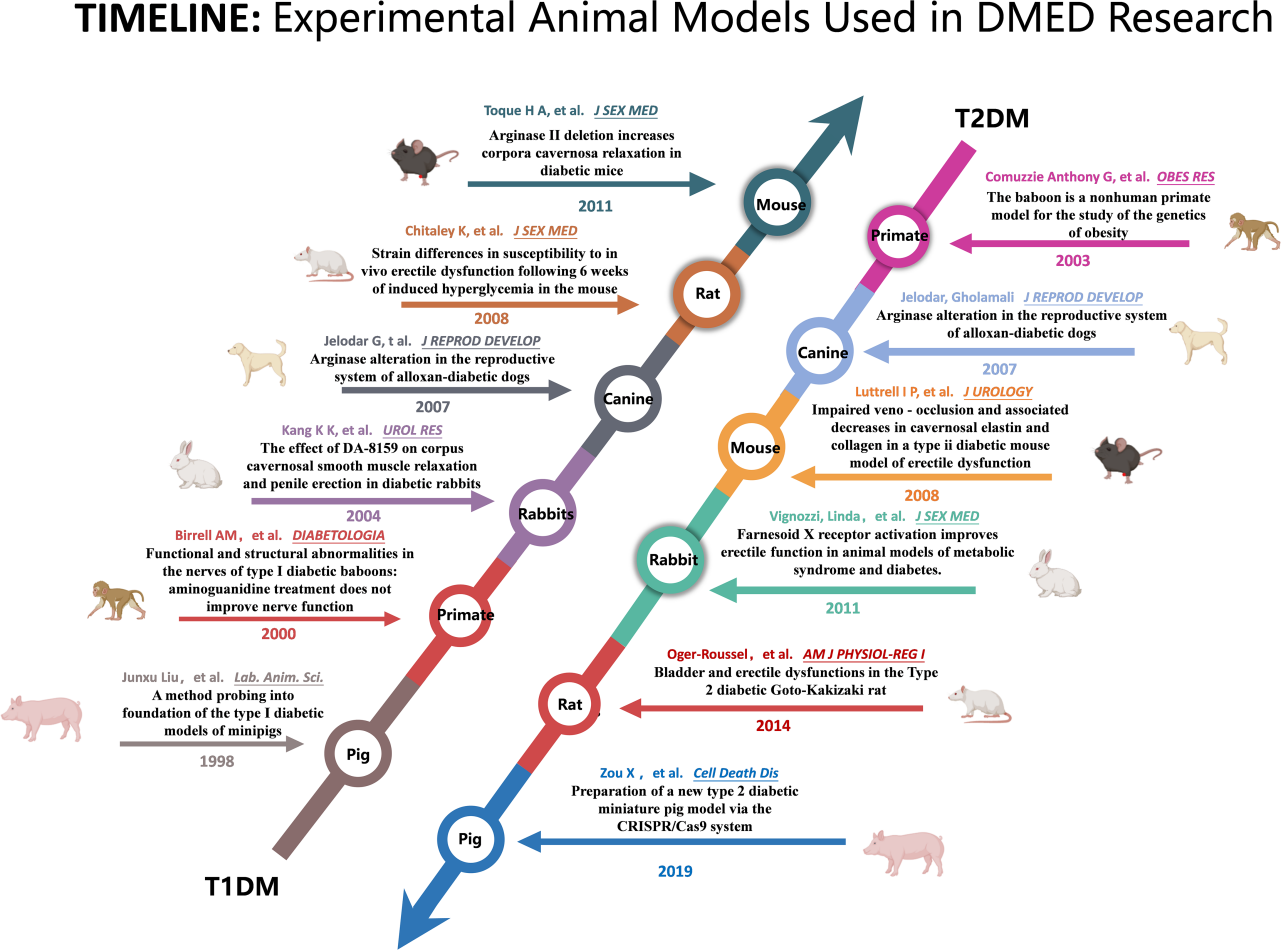
Summary of the key literature on the use of animal models in research on diabetic erectile dysfunction.

A narrative review summarizes the animal models that can be used for experimental studies of DMED in previous studies, as well as the main advantages and disadvantages of these models, as shown in **Table 1**. The main literature is summarized in **Figure 1**.

**Table 1:** Advantages and disadvantages of described diabetes mellitus erectile dysfunction animal model, and literature source.

DMED models	Common types	Literature source	Advantages of specific model	Disadvantages of specific model
Induced DMED models				
High-dose STZ	TIDM	Gvazava, I G et al, 2020 (73)	High-dose STZ destroys pancreatic β-cells, and the method is simple with high reproducibility	STZ is toxic to organs, and animal models differ from the mechanism of diabetes in humans
ALX	TIDM	Srinivasan K and Ramarao P, 2007 (74)	ALX selectively destroys pancreatic β-cells, and the method is simple and easy to perform. It has lower toxicity compared to STZ	Constructing an animal model requires a longer time. ALX can only destroy pancreatic β-cells, also by species Absent of autoimmune processes
Low-dose STZ	T2DM	Xiang X et al, 2010 (40)	low toxicity and is more in line with the characteristics of human diabetes than the high-dose STZ model	Diabetes is reversible, but the stability of the model is low, and the dose selection and experimental conditions need to be more strictly controlled
Spontaneous DMED models				
BB-DP rat	T1DM	Rees DA and Alcolado JC, 2005 (75)	Spontaneous T1DM features, stable genetic predisposition, and high reproducibility	The cost is high, the operation is complex, and there is a high mortality rate in animals
NOD mouse	T1DM	Anderson MS et al., 2005 (76)	Metabolic features similar to human T1DM and does not require insulin (easy to maintain)	marked sexual dimorphism (only 60% of males develop DM)
ZDF rat	T2DM	Srinivasan K and Ramarao P, 2007 (74)/ Augstein P et al., 2009 (57)	Mechanisms close to human T2DM: Metabolic syndrome, hyperlipidemia, and hypertension; it has high stability and reproducibility	There are gender differences, and the cost is higher. There is a higher mortality rate due to ketoacidosis
KK-Ay mouse	T2DM	Iizuka Y et al., 2022 (60)	High genetic stability and exhibits significant metabolic features of T2DM	Results from a monogenic mutation, are expensive and require insulin injections.
ob/ob and db/db mouse	T2DM	Srinivasan K and Ramarao P, 2007 (74)/Lindstrom P et al., 2010 (62)	The phenotype of metabolic syndrome, similar to the human condition Pancreas also shows similar patterns	Dependent on a monogenic mutation in leptin or its receptor. Loss of human heterogeneity. Expensive. Limited insulin required
NSY mouse	T2DM	Shibata M et al,1980 (64)	Cost-effective, with a phenotype of metabolic syndrome similar to the human condition	The modeling process is time-consuming and the disease incidence varies among individuals.
OLETF rat	T2DM	Kawano K et al,1991 (51)	Genetically stable, with a development process similar to human diabetes	Low incidence, slow development of DM, mild obesity, and high genetic variability
GK/IRS-1-KO mouse	T2DM	Galli J et al., 1996 (56)	Obvious metabolic abnormalities, which develop into T2DM under dietary conditions	Low genetic heterogeneity, lacking the complexity of human type 2 diabetes, and expensive
MKR transgenic mouse	T2DM	Wojtaszewski JF et al., 2001 (66)	Cost-effective, easy to operate, with impaired insulin signaling pathway	Genetically homogeneous, with a long modeling time, and not suitable for large-scale use
Gene editing mouse model in DMED				
PEDF KO mouse	Not mentioned	Che D, et al; 2020 (67)	Demonstrated the direct binding of PEDF(pigment epithelium-derived factor); with Hsp90β on the membrane, and that Hsp90β-Ab blocks the effect of PEDF	The search for the PEDF receptor is still not very advanced.
NOX1 KO mouse	Not mentioned	Alves-Lopes et al., 2016 (69)	Use the mechanism that dysregulation in NOX1-derived ROS and Nrf2-antioxidant system contributes to oxidative stress	Have not determined which signal is responsible for NOX1 activation in IPA VSMCs stimulated with HG (high-sugar) or whether NOX1 is the primary point or is activated by a preceding signal-induced
CAV-1 KO mouse	T2DMED	Parikh et al., 2017 (68)	The use of KO mice and novel hemodynamic techniques are the strengths.	Lacking of direct evaluation of penile hemodynamics in T2DM mice.
Ninj1 KO mouse	Not mentioned	Yin et al., 2014 (70)	Through Ninj1-KO mice have found a pathway that therapeutic endothelial and neural regeneration may cure diabetic ED.	It did not explain how Ninj1 regulates the expression of Ang1 and Ang2 or how the Ang1–Tie2 signaling pathway is involved in the regulation of neurotrophic factor expression mediated by Ninj1-neutralizing antibody or by Ninj1 siRNA.
Arg-II KO mouse	T1DMED	Toque et al., 2010 (71)	Using KO mice to find the connection with Arg-II and nitrergic nerve relaxation responses in CC (corpus cavernosum).	Further studies are needed to determine whether other phosphorylation sites of eNOS are involved in the penis
iNOS KO mouse	T1DMED	Ferrini et al., 2010 (72)	Using KO mice to show the inactivation of the iNOS gene exacerbates corporal fibrosis in diabetes.	Has not been further studied other than for its immunohistochemical detection in some patients with diabetes

DMED, diabetes mellitus erectile dysfunction; T1DM, Type 1 Diabetes Mellitus; T2DM, Type 2 Diabetes Mellitus; STZ, streptozotocin; ALX, Alloxan; BB-DP rat, Biobreeding Diabetes Prone rat;NOD mouse, Non-Obese Diabetic mouse; ZDF rat, Zucker diabetic fatty rat; KK-Ay mouse, KK-Ay/TaJclmouse; ob/ob mouse, leptin-obese; db/db mouse, Leprdb/Leprdb; NSY mouse, Nagoya-Shibata-Yasuda mouse; OLETF rat, Otsuka Long-Evans Tokushima Fatty; GK/IRS-1-KO mouse, Goto-Kakizaki/IRS-1 knockout; MKR transgenic mouse, Muscle-Specific Kinase Receptor transgenic mouse

### Evolution of animal models in diabetic erectile dysfunction research

Research examining the physiological and pathological mechanisms of erectile dysfunction has come a long way. In the early studies (1960-1990), researchers predominantly utilized larger animal models, such as dogs, rabbits, monkeys, cats, and others (9).

Dogs, as large experimental animals, share similar physiological characteristics with humans, particularly in the way they receive sexual stimuli, which is predominantly visual. They were initially employed in the investigation of sexual dysfunction; however, due to issues such as high cost and limited longitudinal repeatability, they gradually became less prevalent in conventional ED research. In the past, some studies utilized dogs as models to assess and verify the efficacy of PDE5 inhibitors, resulting in reliable experimental data (10, 11). Nonetheless, the utilization of dogs for constructing animal models of DMED and conducting research is exceedingly uncommon. Only a handful of researchers have employed these animals in experimental studies. The pertinent literature solely reports that DM can heighten arginase activity in the viscera of canines and might lead to erectile dysfunction in diabetic patients (12).

Rabbits are frequently utilized in research related to ED and DMED. The commonly used breed is New Zealand white rabbits. Due to their ease of care and the simplicity of accessing external genitalia, they are often used for physiological function experiments, such as post-injection observation of vasoactive drugs (13). Many studies have compared rabbit and rat models as the foundation for *in vitro* and *in vivo* investigations. A classic drug-induced DMED model in rabbits involves the injection of Alloxan (ALX), which pharmacologically destroys the β-cells of the islets, halts insulin secretion, and induces experimental alloxan diabetes in the animals. As a result, the integrity of corpus cavernosum vascular endothelial cells and smooth muscle cells is altered, leading to increased glycation products and oxidative stress, reduced NOS activity, and induced ED in the animals (14, 15).

Pigs are anatomically and physiologically more akin to humans than other animals, and miniature pigs are commonly used in diabetes models. Liu et al. (16) intravenously injected 5% Alloxan at 200 mg/kg in miniature pigs, resulting in symptoms such as hyperglycemia, glucosuria, and impaired glucose tolerance, indicating that intravenous Alloxan injection can establish a type I diabetes model in miniature pigs. Wu Yanjun et al. (17)employed a high-fat and high-sugar diet combined with low-dose STZ to create a T2DM model in Guangxi Bama miniature pigs. The pigs in this model exhibited apparent and stable characteristics, all manifesting symptoms like insulin resistance and impaired glucose tolerance.

When it comes to animal models for research, using monkeys as a representation of primates can lead to more accurate findings. However, due to the limited availability of primates and the high costs associated with their use, especially with the emphasis on animal protection and the 3R principle (reduction, refinement, and replacement), primates are not frequently used in research related to ED and DMED models.

In the past three decades, rodents have become the preferred choice for animal models in experiments, surpassing other species (9). There are several compelling reasons for this shift: firstly, rodents offer economic and logistical advantages over larger animals in terms of procurement, housing, maintenance, and disposal; secondly, extensive evidence supports that rodent models possess tissue morphology and functional characteristics similar to those of humans; thirdly, anatomically isolating cavernous nerves (CNs) and conducting follow-up studies are easier in rodent models; fourthly, rodent models can effectively measure quantitative changes in intracavernous pressure (ICP) following electrical stimulation around the corpus cavernosum and pelvic nerves; and finally, the relatively short lifespan of rodents enables longitudinal studies to assess disease states and the effects of therapeutic interventions over a compressed timeframe, allowing monitoring of the biological responses of the animals (18, 19).

### Preparation methods, reliability, and know-how of diabetic erectile dysfunction animal models

Rodents have been widely used since the 1990s to study the physiology and pathology of erectile dysfunction, particularly in creating erectile dysfunction models related to metabolic diseases. This section will focus on the preparation methods of DMED models in rats and mice.

### Type 1 diabetic erectile dysfunction model

**Figure 2:**
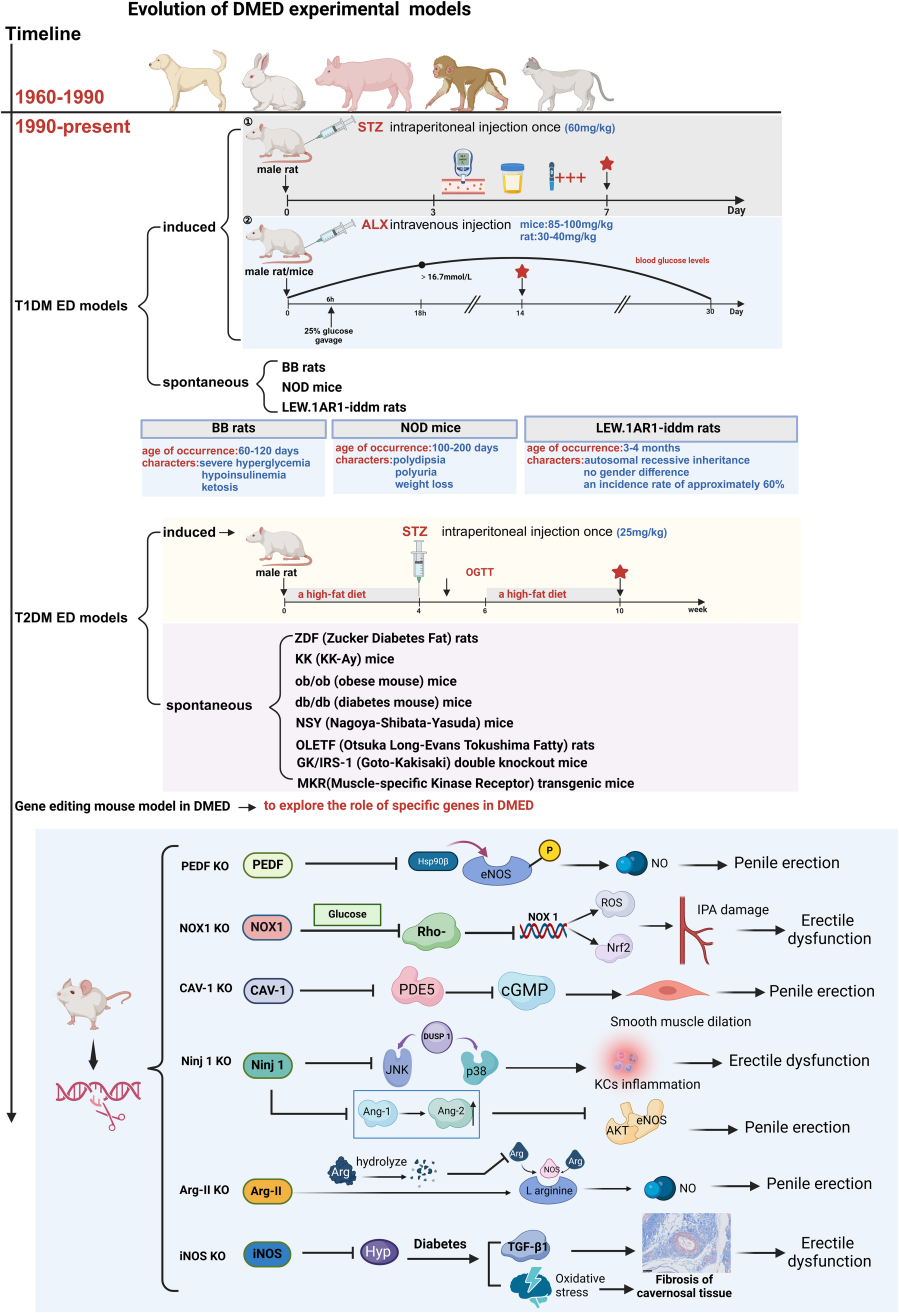
Evolution of DMED experimental models. T1DM, Type 1 Diabetes Mellitus; T2DM, Type 2 Diabetes Mellitus; STZ, streptozotocin; ALX, Alloxan; BB-DP rat, Biobreeding Diabetes Prone rat; NOD mouse, Non-Obese Diabetic mouse; ZDF rat, Zucker diabetic fatty rat; KK-Ay mouse, KK-Ay/TaJcl mouse; ob/ob mouse, leptin-obese; db/db mouse, Leprdb/Leprdb; NSY mouse, Nagoya-Shibata-Yasuda mouse; OLETF rat, Otsuka Long-Evans Tokushima Fatty; GK/IRS-1-KO mouse, Goto-Kakizaki/IRS-1 knockout; MKR transgenic mouse:Muscle-Specific Kinase Receptor transgenic mouse; Kcs, Kupffer cells.

Most studies on DMED are conducted using animals with T1DM, and the preparation of T1DM models mainly includes induced and spontaneous methods, a brief description is shown in **Figure 2**.

#### Inducible animal models

STZ is the most commonly used inducer for this model. The nitrosourea structure of this drug can selectively damage the islet β cells, leading to islet remodeling and fibrosis. Key factors for the success of the model are the STZ injection dose and method. A large injection dose will completely damage the pancreas islets, resulting in absolute insulin deficiency and the formation of type 1 diabetes (73). The intraperitoneal injection is the usual method, which is cost-effective and easy to perform. However, accidental injection into the intestinal tract may lead to the death of the animal (20). Previous research reports have identified rats as the preferred model animals for STZ, as mice are not highly sensitive to STZ, leading to potentially significant differences within the group (21). Additionally, compared to mice, rats are more advantageous for electrophysiological assessments of erectile function, such as intracavernosal pressure monitoring. The STZ injection dose typically ranges from 40 to 75 mg/kg, with studies demonstrating that a single large-dose injection yields the best results. Small-dose injections are more likely to cause insulitis, while a dose of 75 mg/kg has been reported to effectively induce diabetes in rats with consistent outcomes (22). Therefore, experiments often use a dose of 60-75 mg/kg for intraperitoneal injections in rats.

Usually, 2-3 days after the STZ injection, blood glucose levels and urine glucose levels are measured using test strips for three consecutive days. After a week, the tests are repeated. If the rat’s blood sugar is ≥16.7mmol/L and urine sugar is ≥3+, accompanied by symptoms such as polydipsia and polyuria, it indicates the successful establishment of the diabetes model. Approximately six months into the experiment, the cavernous bodies of STZ-induced chronic diabetic rats experienced a significant decrease in both smooth muscle and endothelial cell density (23). A previous study also observed selective degeneration of nitrergic nerves in the corpus cavernosum of diabetic rats, which explains the significantly reduced neuronal nitric oxide synthase (nNOS) expression detected (24). Bivalacqua et al. (25)also found reduced endothelial nitric oxide synthase (eNOS) expression in the STZ model, and both the reduction of NO level and the erectile response could be improved by overexpressing the eNOS gene.

ALX is another commonly used model inducer (74). It is a cytotoxic substance that destroys islet β cells by producing superoxide free radicals, resulting in impaired insulin synthesis and reduced secretion. Its effect may be related to interference with endosomal zinc metabolism. The blood glucose response caused by ALX injection can be divided into three phases: initially, blood sugar rises and lasts for about 2 hours; then, hypoglycemia occurs for approximately 6 hours due to the release of residual insulin from the islet β cells; finally, persistent hyperglycemia begins after 12 hours. The key to the model’s success lies in the injection method and post-processing. Intravenous injection yields better results than intraperitoneal injection, and the speed of intravenous injection is generally considered faster (26). Studies have shown that both rats and mice can be used to create models with ALX, with a higher injection rate of 85-100mg/kg for mice and a concentration of 1%-3% for rats. The intravenous injection dose is 30-40mg/kg (27, 28). Male rats or mice are preferred, and they should fast without food and water for 12 hours before modeling to increase animal sensitivity to ALX. It should be noted that guinea pigs are resistant to ALX and cannot be used as the target of this product (29).

Based on the pharmacological effects of ALX, 6 hours after the injection of this product, it is recommended to use a 25% glucose solution for intragastric administration to significantly reduce animal mortality. Usually, 18 hours after drug injection, the blood sugar of rats will rise above 16.7mmol/L, accompanied by typical symptoms such as polydipsia and polyuria, allowing the type 1 diabetes model to form in about 2 weeks. Studies have found that in alloxan-induced DMED models, the reactivity of cavernous smooth muscle to prostaglandins is impaired (30), the expression and synthesis activity of eNOS protein are reduced (31), and vascular structural changes are promoted, which decrease blood supply and affect hemodynamics, leading to erectile dysfunction (32). This model can effectively simulate changes associated with diabetic complications. However, many studies have shown that after 1 month of ALX injection, blood glucose in some rats will return to normal levels, which may be due to the compensatory effect of rat islets (33). Therefore, ALX modeling is more suitable for short-term observation.

### Spontaneous animal models

#### BioBreeding rats

BB rats are spontaneous, stable hereditary T1DM animals derived from Wistar rats (75). The pathogenesis is associated with pancreatitis induced by autoimmune destruction of islet β cells and insulin deficiency. Diabetes in these rats manifests suddenly at about 60-120 days of age (with reported earliest onset at 48 days), resulting in severe hyperglycemia (252-732mg/dl), hypoinsulinemia (0-1ng/ml), and ketosis (6-13mM). Due to their striking similarity to human juvenile diabetes, BB rats serve as an ideal animal model for spontaneous type 1 diabetes, effectively simulating the occurrence, development, and disease outcome of type 1 diabetes without the involvement of external factors. Studies have shown that BB rats exhibit diffuse neuropathy without significant vascular lesions, indicating the importance of neuropathic processes in DMED development (34). Additionally, it has been confirmed that BB rats exhibit reduced penile nNOS activity, decreased smooth muscle/collagen ratio and smooth muscle content, and increased apoptosis (35).

#### Non-obese diabetic mouse mice

NOD mice are inbred hybrids of cataract-susceptible substrain diabetic mice derived from JCL-ICR strain mice (76). The onset of diabetes in NOD mice is sudden, usually occurring at 100-200 days of age. Diabetes manifests as polydipsia, polyuria, weight loss, and significantly elevated blood sugar. Without insulin treatment, the animals cannot survive for a month and typically succumb to ketosis (36). The pathogenesis of diabetes in NOD mice may be associated with immune-mediated islet inflammation, making them a valuable model for spontaneous autoimmune-related type 1 diabetes (37). The incidence of diabetes in NOD mice is gender-dependent, with female mice experiencing a significantly higher incidence and earlier onset. While this makes them a suitable model for studying the pathogenesis of type 1 diabetes in women, they are not ideal for research on DMED.

#### LEW.1AR1-iddm/Ztm rats

The LEW.1AR1-iddm/Ztm rat is a spontaneous animal model of type 1 diabetes resulting from a mutation within the inbred strain LEW.1AR1 of major histocompatibility complex recombination (38). It exhibits an autosomal recessive inheritance of genetic susceptibility with an incidence rate of approximately 60% and no gender difference. The pathogenesis of diabetes in LEW.1AR1-iddm/Ztm rats primarily involves the immune system attacking insulin-producing β-cells, leading to decreased insulin secretion and hyperglycemia. However, symptoms of diabetes often appear after 3-4 months of age (39). Due to its limited genetic heterogeneity and late onset, LEW.1AR1-iddm rats are infrequently used to establish animal models of DM. However, there is scarce literature reporting on the establishment of DMED models using LEW.1AR1-iddm rats.

### Type 2 diabetic erectile dysfunction models

#### Inducible animal models

ED secondary to T2DM is the most prevalent manifestation, accounting for approximately 90% of DMED patients. Thus, the preparation of T2DM ED models has become a critical research tool. Commonly used T2DM ED models are categorized into two types: induced and spontaneous, a brief description is shown in **Figure 2**.

Type 2 diabetes is characterized by decreased insulin secretion and insulin resistance. To induce a T2DM animal model closely resembling humans, researchers typically administer a small dose of STZ intraperitoneally to destroy a portion of islet β cells and simultaneously feed the animals with a high-fat diet, which reduces the sensitivity of peripheral tissues to insulin. STZ is commonly used at a dose of 25mg/Kg for intraperitoneal injection (40). Studies have reported that after feeding male Wistar rats with a high-fat diet for 4 weeks, STZ was intraperitoneally injected at 25 mg/Kg body weight once, and an oral glucose tolerance test was performed 2 weeks later. Rats with blood glucose levels greater than 7.0 and 11.0 mmol/L at 0 and 120 min were selected and fed with a high-fat diet for 4 weeks to establish an induced type 2 diabetes rat model (41). Another research administered a high-fat diet to male Sprague-Dawley (SD) rats for 4 weeks, followed by intraperitoneal injection of STZ at a dose of 27.5 mg/Kg body weight. After one week, fasting blood glucose levels > 11.1 mmol/L were used as a standard for modeling. The model rats exhibited polydipsia, polyphagia, and polyuria, along with increased blood sugar volatility (15-20 mmol/L), obesity, insulin resistance, and hyperlipidemia (42). Italiano, G (43). et al. studied the effect of diabetes on erectile function in a T2DM animal model induced by a small dose of SZT.

After the model of T2DM is established, the presence of erectile dysfunction (ED) is typically assessed and verified through the following methods: Electrophysiological methods are used to measure penile erectile function, such as assessing the response of the corpus cavernosum and dorsal nerve of the penis. Additionally, behavioral characteristics can be observed by recording the frequency and quality of erections when in contact with a female rat. Furthermore, subcutaneous injection of apomorphine is also commonly used to detect the presence of ED in diabetic animal models. This method is based on the central action of apomorphine, which induces erections by stimulating dopamine receptors, thereby evaluating the erectile response of the animals. These methods can effectively assess the severity of ED in T2DM. Currently, the T2DM model induced by low-dose SZT in rats is one of the most commonly used animal models for studying DMED (44–46).

#### Spontaneous animal models

Spontaneous type 2 diabetes animal models are mostly inbred purebred mice with a predisposition to spontaneous diabetes, aiming to obtain offspring with stable genetic phenotypes after mating. These animals are fed under specific feeding conditions. Commonly used spontaneous type 2 diabetes model mice mainly include db/db mice, OLETF rats, ZDF rats, KK mice, and ob/ob mice.

#### Leptin receptor-deficient mice (db/db mice)

db/db mice are spontaneously diabetic mice derived from C57BL/KsJ strain, and they also exhibit autosomal recessive inheritance. At one month of age, these mice start to exhibit bulimia and weight gain, and subsequently develop hyperglycemia, hyperinsulinemia, and elevated glucagon levels. The mice generally die within 10 months, with most of them succumbing to ketosis (47). db/db mice are common animal models of T2DM, and they are frequently used to explore DMED-related mechanisms. For instance, Luttrell, IP (48) et al. investigated the effect of altered vascular reactivity and veno-occlusive disease on erectile function in db/db mice; Nunes, KP (49) et al. confirmed the beneficial effect of soluble guanylyl cyclase stimulator BAY 41-2272 on impaired penile erection in db/db^-/-^ type II diabetic and obese mice.

#### Otsuka Long-Evans Tokushima fatty rats

OLETF rats are the offspring of Long-Evans and Tokushima fat rats and are used to study the pathogenesis and treatment of T2DM (50, 51). These rats initially exhibit hyperphagia and obesity, leading to hyperinsulinemia and hyperglycemia. At approximately 18-25 weeks of age, obvious symptoms of T2DM appear, accompanied by visceral fat accumulation and pronounced insulin resistance (52). Kataoka, T et al. used OLETF rats to study the effectiveness of androgen replacement therapy (ART) in improving erectile dysfunction in T2DM animal models and evaluated the levels of NO and inflammatory factors in the process, the results showed that ART suppressed inflammation in rats with T2DM and metabolic disorders and improved their vascular endothelial and erectile functions (53). However, OLETF rats have some drawbacks as a T2DM model, including high genetic heterogeneity, low incidence of DM, slow progression, and less obesity.

#### Goto-Kakisaki (GK/IRS-1) double knockout mice

GK/IRS-1 double knockout mice were generated by crossing diabetes susceptibility gene GK mice with insulin receptor substrate 1 (IRS-1) knockout mice. These mice were once considered one of the best animal models for studying genetic susceptibility to T2DM in non-obese individuals. Carneiro, FS confirmed that the erectile dysfunction in GK/IRS-1 double gene knockout mice is related to the decrease in eNOS phosphorylation level at Ser1177 (54). Additionally, Oger-Roussel, S (55) et al. confirmed the relationship between bladder dysfunction and erectile dysfunction through GK/IRS-1 double gene knockout mice and demonstrated that sildenafil can effectively improve erectile function. However, compared with human diseases, GK/IRS-1 double gene knockout mice have lower genetic heterogeneity and lack the complexity of human type 2 diabetes (56).

#### ZDF rats

ZDF rats are Zucker diabetic obese rats, which share most of the characteristics of obese Zucker rats, including obesity, insulin resistance, abnormal glucose tolerance test, hyperinsulinemia, hyperleptinemia, hyperlipidemia, etc. Offspring ZDF rats with stable inheritance were obtained through the inbreeding of some Zucker rats born with insulin resistance. Although the blood sugar was not significantly elevated, the serum triglyceride-free fatty acid level was high (57–59). Despite being a common animal model of DM, limited studies are using ZDF rats to investigate the mechanism of DMED.

#### KK (KK-Ay) mice

KK mice are a T2DM mouse model with mild obesity bred by Japanese researchers. After screening, they are crossed with C57BL/6J mice. The inbreeding process results in KK mice with obesity genes and yellow fat referred to as KK-A(y) mice. These mice exhibit symptoms of obesity and diabetes at approximately 5 weeks after birth, followed by degranulation and glycogen degeneration of islet cells, cell hypertrophy, cell vacuolization, and fatty changes (60). KK-A(y) mice are not typically the most common models for studying diabetic ED. However, some literature has reported that mogroside may help prevent erectile dysfunction and reproductive damage in KK mice by inhibiting the AGEs/RAGE/p38MAPK/NF-κB pathway (61).

#### Obese mouse (ob/ob) mice

ob/ob mice are a typical animal model of type 2 diabetes with obesity and hyperglycemia. This model belongs to autosomal recessive inheritance and is widely used both domestically and internationally. In these homozygous mice, spontaneous obesity, hyperglycemia, glycosuria, and hyperinsulinemia develop at an early stage (62). Without fasting, blood sugar levels are typically higher than 16.67mmol/L (63). Based on the aforementioned characteristics, ob/ob mice are highly suitable for studying obesity-related type 2 diabetes and its complications. However, it is regrettable that there have been no reports to date on the use of this mouse model for investigating the mechanisms of DMED.

#### Nagoya-Shibata-Yasuda mice

NSY mice are a moderately obese spontaneous model of T2DM established by selectively breeding non-diabetic JcI: ICR mice with glucose intolerance (64). These mice spontaneously develop diabetes in an age-related manner, with a cumulative incidence of T2DM reaching 98% in male mice and 31% in female mice at 48 weeks of age (65). However, the long time required for NSY mice to develop diabetes does not aid the study of DMED-related secondary consequences and their underlying mechanisms of action, resulting in limited literature reports using NSY mice.

#### Muscle-specific kinase receptor transgenic mice

MKR transgenic mice are an experimental model in which insulin receptor substrate-1 (IRS-1) is knocked out in muscle tissue. These mice are widely used to study diabetes and insulin signaling pathways. Insulin binds to IRS-1, promoting its phosphorylation, and activates various signal transduction molecules, such as Phosphoinositide 3-Kinase (PI3K), Protein Kinase B (Akt/PKB), and Mitogen-Activated Protein Kinase (MAPK), to mediate the biological effects of insulin. The lack of IRS-1 leads to abnormalities in the insulin signaling pathway in muscle tissue, resulting in reduced responsiveness of muscles to insulin, leading to symptoms such as hyperglycemia and insulin resistance, similar to diabetes (66). However, the phenotype of MKR transgenic mice differs from that of human T2DM patients, and there are issues with the high cost of feeding, leading to their gradual abandonment. Consequently, there are nearly no reports in the literature on the use of MKR transgenic mice to study DMED.

### Gene editing mouse model in DMED

As research progresses, an increasing number of researchers have started editing specific genes to elucidate their roles in the onset and development of DMED. Most of these studies involved the knockout of particular genes in mice, and the functions of each gene are detailed in **Figure 2**. Che et al. (67) demonstrated, using pigment epithelium-derived factor (PEDF) knockdown mice, that high levels of PEDF impair erectile function by inhibiting Hsp90β-mediated eNOS phosphorylation. They also proposed that PEDF might serve as a novel therapeutic target for diabetic erectile dysfunction. Parikh et al. (68) used Caveolin-1 (Cav-1) knockout mice to confirm that high expression of Cav-1 can effectively improve erectile dysfunction caused by DM. Alves-Lopes et al. (69) utilized NADPH oxidase 1 (NOX1) knockout mice to confirm that high glucose can downregulate Rho kinase in vascular smooth muscle cells, leading to increased NOX1-derived Reactive Oxygen Species (ROS) and NF-E2-related factor 2 (Nrf2) systems. This impairment of the internal pudendal artery (IPA) function in DM subsequently results in erectile dysfunction. Yin et al. (70) employed nerve injury factor (Ninjurin 1) knockout mice to inhibit the Ninj1 pathway in diabetes. This intervention successfully restored erectile function by promoting angiogenesis and nerve regeneration. Toque et al. (71) discovered that arginase (Arg-II) subtype-deficient mice exhibited improved relaxation function of cavernous smooth muscle in a STZ-induced diabetes model. On the other hand, Ferrini et al. (72) confirmed the anti-fibrotic, anti-oxidative, and smooth muscle protective effects of inducible nitric oxide synthase (iNOS) in the corpus cavernosum using the iNOS KO/STZ mouse model. Gene knockout or transgenesis can be employed to investigate the impact of nearly any specific molecular mechanism on DMED. 

## Strengths and limitations

### Strengths

The use of rodent models in DMED research offers significant advantages, including cost-effectiveness, physiological similarities to humans, and the ability to conduct longitudinal studies within a compressed timeframe. Advanced genetic techniques, such as clustered regularly interspaced short palindromic repeats associated protein 9 (CRISPR-Cas9), further enhance their utility by enabling the exploration of molecular mechanisms and targeted therapies.

### Limitations

However, limitations must be acknowledged. Rodent models cannot fully replicate the complexities of human sexual behavior, as their erectile responses are primarily driven by olfactory cues and vaginal insertion, differing from human visual and auditory stimuli. Additionally, their short lifespan restricts long-term studies. Despite these limitations, rodent models remain invaluable for DMED research, and ethical adherence to the 3Rs principles (reduction, refinement, and replacement) ensures their responsible use.

## Conclusion and future perspectives

In conclusion, while the prevalence of diabetes mellitus and its complications, including erectile dysfunction, continues to rise globally, animal models—particularly rodents—remain indispensable for understanding the pathogenesis and treatment of DMED. Their practicality, cost-effectiveness, and physiological similarities to humans make them a primary choice, despite their inability to fully replicate the psychosomatic complexity of human sexual behavior.

Future research should focus on refining these models to better integrate behavioral components and enhance their translational relevance. Additionally, advancements in genetic editing techniques, such as CRISPR-Cas9, and the integration of omics approaches (genomics, proteomics, metabolomics) will provide deeper insights into the molecular mechanisms of DMED. By prioritizing ethical research and leveraging innovative technologies, the field can move closer to developing targeted therapies and improving clinical outcomes for individuals affected by DMED.
